# Examining the clinical validity of the global psychotrauma screen in refugees

**DOI:** 10.3389/fpsyg.2024.1394014

**Published:** 2024-07-22

**Authors:** Janaina V. Pinto, Christopher Hoeboer, Caroline Hunt, Brian O’Toole, Miranda Olff

**Affiliations:** ^1^Faculty of Medicine and Health, University of Sydney, Camperdown, NSW, Australia; ^2^Amsterdam University Medical Center, Amsterdam, Netherlands

**Keywords:** screening, refugees, trauma, PTSD, MDD, GAD, dissociation, resilience

## Abstract

**Introduction:**

The Global Psychotrauma Screen (GPS) is a brief transdiagnostic screener that covers a broad range of trauma-related disorders as well as risk factors known to influence the course of symptoms.

**Methods:**

We analyzed data from African war refugees in Australia (*n* = 70), including the GPS, the Structured Clinical Interview for DSM-5 Disorders (SCID-5), the Clinician-Administered PTSD Scale for DSM-5 (CAPS-5), and the Brief Resilience Scale (BRS).

**Results:**

Using the Youden’s *J* Index to examine the clinical validity of the GPS subscales measuring PTSD, dissociation, depression, and generalized anxiety disorder (GAD), we found that a PTSD subscale score of 3 or higher, and a depression and dissociation subscale score of 1 or higher, was optimally efficient for detecting a probable diagnosis (Youden’s *J* = 0.76, *J* = 0.72, and *J* = 0.90, respectively) with high sensitivity and specificity. We were unable to test the GPS clinical validity for GAD due to the low GAD occurrence. The GPS resilience item was not related to the total score (*r* = 0.02), indicating low convergent validity for resilience. Risk factors, including current stressors and childhood trauma history, were related to more severe GPS symptom scores, while lack of resilience, social support, and history of mental illness were not.

**Conclusion:**

We conclude that the GPS may be a useful screening tool for PTSD, depression, and the dissociative subtype in refugees.

## 1 Introduction

Mental health challenges that may emerge as a result of exposure to traumatic experiences are not limited to post-traumatic stress disorder (PTSD). Epidemiological studies indicate that most individuals with PTSD meet the criteria for at least one other and, on average, three other psychiatric diagnoses (Brady et al., 2000), with major depressive disorder (MDD) and generalized anxiety disorder (GAD) most frequently reported (Kar and Bastia, 2006; Grant et al., 2008; Choudhary et al., 2012; Bonde et al., 2016; Price et al., 2019; Van der Kolk et al., 2019; LeMoult et al., 2020; Li et al., 2020). Other significant comorbid PTSD diagnoses include substance use disorder (Roberts, 2021; Roberts et al., 2022) and complex posttraumatic stress disorder (CPTSD) (Spikol et al., 2022).

Besides psychiatric disorders, other significant problems have also been documented to follow exposure to traumatic events, such as sleep abnormalities (Leskin et al., 2002; Singareddy and Balon, 2002; Kobayashi et al., 2007; Krakow et al., 2015), substance addiction and misuse (Brown et al., 1999; Jacobsen et al., 2001; Fareed et al., 2013; Debell et al., 2014), dissociation (Vonderlin et al., 2018) defined as out-of-body depersonalization experiences or derealization consisting of alternate perceptions of reality, physical symptoms including cardiovascular, respiratory, musculoskeletal, neurological, and inflammation (Mcfarlane et al., 1994; O’Toole and Catts, 2008; Ryder et al., 2018; Hori, 2019), and self-harm (Dyer et al., 2009; Gratz and Tull, 2012; Dixon-Gordon et al., 2014).

### 1.1 Refugee mental health

Refugees are one of the largest demographics worldwide exposed to severe traumatic experiences. The United Nations Refugee Agency estimated that by mid-2023, there are over 110 million forced displaced people worldwide, 75% of which hosted in low- and middle-income countries (LMICs) (United Nations High Commissioner for Refugees, 2024). In conflict settings, a meta-analysis conducted by the World Health Organization (WHO) estimated that the average prevalence for MDD, anxiety, and PTSD among affected populations was 13% for mild, 4% for moderate, and 5.1% for severe cases (Charlson et al., 2019). However, some groups of refugees and internally displaced persons (IDPs) in LMICs may present significantly higher incidence than others. For instance, a meta-analysis including 10 studies with 5,287 participants in African countries indicated that the pooled prevalence of PTSD was 56.35% for IDPs, and 54.04% for refugees (Andualem et al., 2024). In alignment, the prevalence of PTSD in Liberia was estimated at 48.3% (Galea et al., 2010), compared to 11.8% among Guatemalan refugees in Mexico (Sabin et al., 2003), or 61% among Burmese refugees in Bangladesh (O'Connor and Seager, 2021).

In high-income countries, a comprehensive systematic review including data from 6,743 refugees resettled across seven countries indicate prevalence rates of 9% (99% CI 8–10%) for PTSD and 5% (4–6%) for MDD (Fazel et al., 2005), up to 10 times higher compared to age-matched local populations. Similar to LMICs, studies also show wide ranges of prevalence rates among different cultural groups. For example, among adult Syrian refugees in Norway, prevalence is estimated at 29.7% for PTSD, 30.1% for anxiety symptoms, and 45.2% depression (Nissen et al., 2021). Among African refugees resettled in Italy, one study documented that 79% of a sample met the DSM-5 criteria for PTSD (Barbieri et al., 2019). Interestingly, research examining the relationship between PTSD prevalence at a national level, the frequency of trauma exposure and the overall cultural and socioeconomic vulnerability of countries to adversity found no significant association between PTSD and vulnerability of the country itself (Dückers et al., 2016). Hence, the study found highest PTSD prevalence in developed countries including Canada, the Netherlands and Australia, and lowest in developing nations such as Nigeria, China and Romania (Dückers et al., 2016).

Overall, studies on the prevalence of mental health challenges among refugees demonstrate that ratings can be as elevated across high-income countries and LMICs. Significant discrepancies in results have been attributed to methodological components (e.g., self-report vs., structured interviews, sample size, diagnostic criteria) explaining 12.9% of the variability (Steel et al., 2009). Accounting for these components, meta-analyses estimate global PTSD prevalence rates in refugees at approximately 30% (Steel et al., 2009; Blackmore et al., 2020). Not accounted for, a significant variant in prevalence of PTSD among refugees may also pertain to complex PTSD (CPTSD) (Liddell et al., 2019), postulated to result from prolonged exposure to significant trauma commonly experienced by refugee populations. In addition to PTSD symptomatology, CPTSD is characterized by disturbances in self-organization (DSO), which includes negative self-concept, challenges with relationships, and emotion dysregulation (World Health Organization, 2018). Importantly, among refugee groups, postmigration living difficulties which exacerbates symptom severity have been primarily associated with DSO and CPTSD, rather than PTSD (Hecker et al., 2018; Silove et al., 2018; Tay et al., 2018; Liddell et al., 2019; Schiess-Jokanovic et al., 2021, 2022; Barbieri et al., 2023).

### 1.2 PTSD screeners

Among widely utilized measures to screen for PTSD in refugees with acceptable psychometric properties are the Posttraumatic Stress Disorder Checklist (PCL-5) (Pereira-Lima et al., 2019), the Harvard Trauma Questionnaire (HTQ) (Berthold et al., 2019), the International Trauma Questionnaire (ITQ) (Cloitre et al., 2021), and the Impact of Event Scale (IES) (Weiss, 2007). While there is a notable surge in efforts to adapt PTSD screeners for cross-cultural populations in LMICs (Wilson and Tang, 2007), several limitations exist, including few translations, poor accessibility, length of screeners, and the need for trained administers. Among screeners cited, the ITQ presents the best accessibility for refugee populations, with 32 languages freely available for download on an open-source website^1^, and has been validated in LMICs among vulnerable populations (e.g., Syrian refugees in Lebanon, Vallières et al., 2018), in addition to being reasonably brief and suitable for self-administration. However, a notable limitation of the ITQ, shared with other measures, is its focus on solely assessing PTSD, lacking transdiagnostic capabilities. This lack has compelled agencies working with refugees to use a combination of measures to document complex outcomes of trauma, resulting in lengthier paper-based efforts that, particularly in LMICs, exhaust limited resources.

The absence of a single instrument to measure varied possible consequences of exposure to trauma led to a collaborative effort by the Global Collaboration on Traumatic Stress (GCTS, Schnyder et al., 2017) to develop the Global Psychotrauma Screen (GPS) (Oe et al., 2020; Olff et al., 2020; Frewen et al., 2021; Rossi et al., 2021). In a short screening format, the GPS broadly assesses several potential consequences of traumatic events, including PTSD, complex PTSD, depression, anxiety, sleep problems, self-injurious behavior, dissociation, substance abuse, and other physical, emotional, or social problems. Furthermore, it documents risk factors for the consequences of trauma, including prior stressful events, history of childhood trauma and mental illnesses, lack of social support, and perceived lack of psychological resilience.

Numerous studies conducted across different countries and populations have demonstrated that the GPS is a valid and reliable instrument (Schnyder et al., 2017; Oe et al., 2020; Olff et al., 2020, 2021; Rossi et al., 2020, 2021; Cao et al., 2021; Grace et al., 2021, 2023; Williamson et al., 2021; Hoffman et al., 2022; Marengo et al., 2022; Havermans et al., 2023; Salimi et al., 2023; Bi et al., 2024; Frewen et al., 2024; Koutsopoulou et al., 2024). Research on the psychometric properties of the GPS present evidence of robust reliability, as well as convergent and concurrent validity with measures of trauma-related symptom domains. For instance, in two general population samples of English-speaking participants aged 18 and over, Frewen and colleagues documented strong internal consistency for the 17 GPS symptom items (*α* = 0.94) and a cut-off GPS symptom score of 8 for optimal sensitivity (83%) which refers to the screener’s ability to identify the condition, and relative to specificity (71.1%), indicating that majority of patients without the condition were true negatives (Frewen et al., 2021). These resulted in a sensitivity of minimally 80% and area under the ROC curve (AUC) measuring test accuracy = 0.839 for predicting probable PTSD (Frewen et al., 2021), based on scores on the PCL-5 (Blevins et al., 2015). The researchers also identified a single factor via exploratory factor analysis, indicating that one latent construct underlies a substantial part of the outcomes of trauma (Frewen et al., 2021). This corresponds to the high comorbidity between trauma-related disorders.

In a cross-sectional study examining the psychometric properties of the Japanese version of the GPS, including 58 individuals with trauma history, researchers identified excellent internal consistency for the GPS total scores (*α* = 0.90) (Oe et al., 2020). The GPS total score was highly correlated with the PCL-5, the Patient Health Questionnaire (PHQ-9, Kroenke et al., 1999), a measure for depression, and a self-report GAD measure, the generalized anxiety disorder-7 (GAD-7, Spitzer et al., 2006), (*r* > 0.79) (Oe et al., 2020). In addition, the GPS subscale scores correlated with corresponding domain scales assessing the several potential consequences of traumatic events, showing good concurrent validity (Oe et al., 2020). In a study validating the GPS in a LMIC, Indonesia, researchers found acceptable AUCs (> 0.70) for all probable diagnoses including PTSD, CPTSD, GAD, and MDD, except for insomnia (Primasari et al., 2024).

The GPS has also been used and found reliable in a global study examining the mental health of populations among 7,034 participants from 88 countries and 12 UN regions (*α* = 0.88) (Olff et al., 2021). Moreover, in a large Italian sample, the convergent validity of the GPS was assessed for the GPS symptom total score and the PHQ-9, GAD-7, and the Insomnia Severity Index (ISI, Morin, 1993), all yielding statistically significant correlations (*p* < 0.001) (Rossi et al., 2021). While the accumulated evidence for the GPS is good with demonstrated cross-cultural cultural capabilities, the accuracy of the screener has not been tested among clinical and refugee populations. As refugee numbers continue to rise worldwide (United Nations High Commissioner for Refugees, 2022), health providers in low-resource settings could significantly benefit from a brief and transdiagnostic screening tool.

This study examined the reliability and clinical validity of the GPS among refugees. We hypothesize that the GPS will present adequate efficiency for detecting a probable diagnosis of PTSD, MDD, and GAD with moderately high sensitivity and specificity (>0.7/0.8).

## 2 Materials and methods

### 2.1 Ethics approval, consent and recruitment

GPS data was collected between December 2018 and October 2020 as part of a larger clinical trial in Queensland and New South Wales, Australia. The study was approved by the University of Sydney Human Research Ethics Office and was conducted in compliance with ethics committee approval conditions. All participants voluntarily provided oral and written informed consent at baseline. Participants were recruited in collaboration with refugee-led organizations in Queensland, Australia. A protocol detailing the study methodology has been published elsewhere (Pinto et al., 2022).

### 2.2 Inclusion and exclusion criteria

Inclusion and exclusion criteria were aligned to the aims of the larger clinical trial (see Pinto et al., 2022). Eligibility included trauma-exposed Liberian, Congolese or Sudanese refugees aged 18 and over who migrated to Australia having departed Africa during civil wars, fragile peace periods, or Ebola endemics (1989–2018). Moreover, eligibility for half of the sample (those in the PTSD condition of the original trial) included meeting the diagnostic criteria for clinical or subclinical PTSD, while this was an exclusion criteria alongside the presence of other psychiatric diagnoses for the other half of the sample (those in the control condition of the original trial). All participants were required to have English proficiency. Exclusion criteria included severe medical conditions or genetic disorders, being at acute risk of suicide or homicide, drug or alcohol abuse or dependency in the previous month, current use of psychotropic medication or use within the 2 months preceding the study, and concurrent psychotherapy for PTSD.

### 2.3 Measures

#### 2.3.1 Global psychotrauma screen

The GPS version 1.2 utilized in this study includes 22 questions with a binary yes/no answer format, of which 17 are symptom questions assessing transdiagnostic trauma related symptoms such as posttraumatic stress, DSO, anxiety, depression, sleep problems, dissociation, self-harm, substance abuse, and other physical, emotional, or social problems, alongside five questions assessing risk and protective factors, including other stressful events, childhood trauma, history of mental illness, social support, and psychological resilience. The GPS can produce several scores, including GPS symptoms, defined by the sum score of all symptom items (range 0–17) reflecting the overall transdiagnostic burden; risk and protective factors, defined by the sum of all risk and protect items (range 0–5), as well as subscale scores for PTSD (range 0–5); DSO (range 0–2); CPTSD (range 0–7); anxiety (range 0–2); depression (range 0–2); insomnia (range 0–1); self-harm (range 0–1); dissociation (range 0–2); substance abuse (range 0–1), and other problems (range 0–1). The GPS can be found here: www.global-psychotrauma.net/gps.

#### 2.3.2 Structured clinical interview for DSM-5 disorders (SCID-5)

MDD and GAD were assessed using the SCID-5 (First et al., 2015a), a semi-structured interview for all primary DSM-5 diagnoses. The SCID-5 presents excellent inter-rater reliability, with nearly all kappa values reported being 0.75 or higher, strong clinical validity with diagnostic sensitivity above 0.70, and specificity above 0.80 (Osório et al., 2019). For MDD, the SCID-5 has evidence of good interrater reliability (*κ* = 0.76) and excellent sensitivity (0.96) and specificity (0.85), while for GAD, it has evidence for reasonable interrater reliability (*κ* = 0.61), good sensitivity (0.72) and excellent specificity (0.94) (Osório et al., 2019) The SCID has been widely utilized cross-culturally as a gold standard measure to assess psychiatric conditions, including among Sub-Saharan African refugees (e.g., Tay et al., 2013; Wulfes et al., 2019).

#### 2.3.3 The clinician-administered PTSD scale for DSM-5 (CAPS-5)

Both PTSD and dissociative subtype were assessed using the CAPS-5 (Blake et al., 1995; Weathers et al., 2001, 2018). The CAPS-5 is a structured assessment tool for DSM-5 PTSD diagnosis and symptom severity, containing 30 items, of which 20 correspond to the symptomatology of a single index trauma. Standardized questions and probes assess each symptom using a 5-point severity rating scale ranging from 0 (absent) to 4 (extreme/ incapacitating), in which scores of two or greater are considered clinically relevant. Additional questions assess the onset and duration of symptoms, level of subjective distress, impairment in social and occupational functioning, response validity, symptom severity, and dissociative symptoms to inform a diagnosis of PTSD. Note that the dissociative subtype was established when participants met criteria for both the PTSD diagnosis and dissociation. The diagnostic component of the CAPS-5 has evidence of excellent convergent and discriminant validity, with a strong correspondence with diagnoses made using the CAPS-IV (*κ* = 0.84; Weathers et al., 2018), strong interrater reliability (*κ* = 0.78–1.00), and test–retest reliability (*κ* = 0.83). For the measurement of trauma symptom severity, the CAPS-5 total severity score demonstrated strong convergent validity with the CAPS-IV total severity score (*r* = 0.83), excellent internal consistency (*α* = 0.88), good interrater reliability (ICC = 0.91), and reasonable test–retest reliability (ICC = 0.78; Weathers et al., 2018). The CAPS is a gold standard assessment for PTSD used cross-culturally among refugees and in LMICs (Blackmore et al., 2020). This measure has also been used among Sub-Saharan Africans in validation studies of other PTSD screeners (Renner et al., 2006; Verhey et al., 2018).

#### 2.3.4 Brief resilience scale

Resilience was assessed using the Brief Resilience Scale (BRS, Smith et al., 2008), a six-item self-report measure of subjective resilience, categorized as the ability to bounce back from stress. Each question is answered on a 5-point Likert scale ranging from 1 (strongly disagree) to 5 (strongly agree). The BRS has evidence of good internal consistency *α* = 0.80–0.91, and a one-month test–retest reliability ICC = 0.69 among diverse populations. There are also reports for the BRS of good convergent and predictive validity positively correlating with measures of active coping, positive reframing, and planning (*r* = 0.27–0.42), as well as negatively correlating with a range of poor health-related outcomes such as perceived stress, negative affect, anxiety, and depression (*r* = 0.34–0.60) (Smith et al., 2008).

### 2.4 Data collection

All measures included in this present GPS validation analyses were collected during the study baseline phase. The SCID-5 and the CAPS-5 interviews were administered by a trained master’s level clinician, while the BRS and the GPS were completed by participants as a self-report assessment.

### 2.5 Statistical analyses

Internal consistency of the GPS symptom score and subscales were checked using Cronbach’s alpha, Cronbach’s alpha when item deleted, inter-item and item-total correlations (based on Spearman’s Rho). Cronbach’s alpha >0.75, inter-item correlations between.15 and 0.50 and item-total correlations >0.30 were considered adequate. Clinical validity of the GPS subscales measuring PTSD, dissociation, depression, and GAD was evaluated with respective diagnostic reference standards for PTSD and the dissociative subtype (CAPS-5), and for MDD and GAD (SCID-5). We determined the optimal cut-off with the Youden index^2^, thereby maximizing the sum of the sensitivity and specificity. For the dissociative subtype, we first established the optimal cut-off for PTSD and dissociation, and then combined these two. Thus, scoring above the cut-off for both PTSD and dissociation was scored as a positive screen for the dissociative subtype. We reported corresponding sensitivity, specificity, positive predictive value^3^, and negative predictive value^4^, including 95% confidence intervals (note that these were corrected for the case–control design), likelihood ratios and receiver operating curves, including the area under the curve. We also tested the convergent validity of the GPS resilience item with the BRS total score with a point-biserial correlation coefficient. Finally, we tested whether risk factor items of the GPS were related to GPS symptom scores via a series of linear regression analyses. Analyses were performed in SPSS (IBM Corp., 2019) and Team RC (2020) using R packages OptimalCutpoints and cutpointsr.

## 3 Results

### 3.1 Recruitment results

At intake, 100 individuals were screened, among which 20 did not meet the study’s inclusion criteria (*n* = 7 controls with psychiatric conditions, *n* = 6 reported substance abuse, *n* = 4 had poor English fluency, *n* = 2 were unable to be reached after initial intake, and *n* = 1 was outside of age range). At baseline, 80 participants were fully assessed, with five not enrolled (*n* = 4 due to busy schedules, and *n* = 1 reason undisclosed). Among the final 75 individuals who enrolled, five participants opted out of the study (*n* = 3 due to busy schedules, *n* = 1 alcohol abuse disclosure, *n* = 1 loss of interest), consenting that their data be removed.

#### 3.1.1 Sociodemographic characteristics

The final sample included 25 Sudanese, 23 Congolese, and 22 Liberian participants aged 18–54 (*M* = 33.64, SD = 10.54), representing 26 African ethnic groups. In total, there were 31 males and 39 females. Regarding education levels and employment, 14.8% completed primary or high school, 50.8% completed technical or vocational training, 34.3% completed university undergraduate or postgraduate degrees, 55.4% were currently employed, 21.5% were unemployed, and 23.1% were students. Less than half (34.8%) of the participants were married. In total, 44.3% of the participants were born during active war or conflict, 15.7% were born inside a refugee camp or settlement, and 67% reported residing in one at some stage.

On average, participants fled war and conflict 2.11 (*SD* = 1.40) times before migrating to Australia and had been resettled for 10.57 years (SD = 5.29; Range: 1–21). All participants migrated to Australia on humanitarian visas and had access to improved quality of life and resources offered by the Australian government, including education, subsidized housing in districts where refugee communities can live nearby one another, and health care. However, the PTSD group (in the main trial) reported significantly higher levels of post-migration living difficulties (*M* = 28.39, *SD* = 14.53) than the control group (*M* = 16.38, *SD* = 15.11), *t*(63) = 3.26, *p* = 0.002, according to the Post-Migration Living Difficulties (PMLD) scale. Moreover, there was a positive correlation between PTSD symptom severity and PMLD scores *r_s_*(68) = 0.476, *p* < 0.001.

Mean scores across all instruments were predominantly higher among female participants, as detailed in Table 1.

**Table 1:** Mean scores.

Measure	*N*		Mean		Std. deviation	
	*F*	*M*	*F*	*M*	*F*	*M*
GPS symptoms	39	31	6.8	4.5	5.0	3.6
BRS total	31	27	19.4	18.6	3.6	4.4
CAPS-5 severity total	39	31	23.0	14.4	21.8	16.6
CAPS-5 depersonalization severity	39	31	0.7	0.5	1.1	1.1
CAPS-5 derealization severity	39	31	0.7	0.5	1.1	1.0
SCID-5 GAD diagnosis	35	30	1.1	1.1	0.4	0.3
SCID-5 MDD diagnosis	37	29	1.6	1.1	0.9	0.4

F, female; M, male.

#### 3.1.2 Traumatic event exposure

According to the CAPS-5 criteria A, traumatic events across the total sample were experienced directly (91.4%), witnessed (84.3%), and learnt about (100%). In addition, participants reported being repeatedly exposed to aversive details of others’ trauma within their families and communities (85.7%). Threat types included life threat to self (91.4%), life threat to others (84.3%), serious injury to self (48.6%), serious injury to others (84.3%), sexual violence to self (37.1%), and sexual violence to others (68.6%). In total, 77.1% of participants reported experiencing early life stress (ELS) under the age of 12, while 30% also reported ELS between the ages of 13–19. While all participants were primarily war refugees, they identified their worst traumatic event as war (87.1%), sexual assault (7.1%), domestic violence (4.3%), and one participant reported a motor vehicle accident (1.4%) without significant impairment.

### 3.2 Global psychotrauma screen

**Figure 1:**
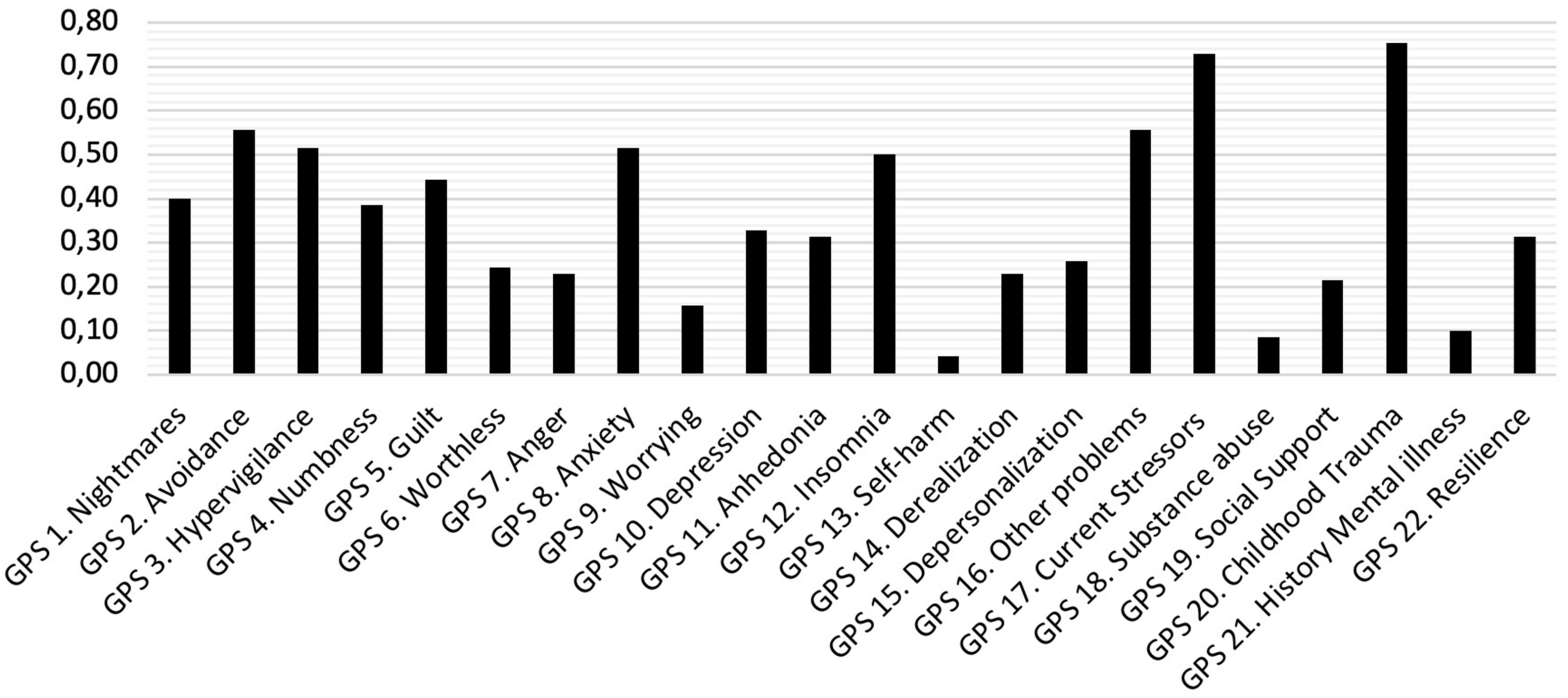
Item endorsement of the GPS.

Item endorsement of the GPS is shown in Figure 1. Items about self-harm and substance abuse were rarely endorsed (<10%). Notably, two risk factors, childhood trauma and current stressors were highly endorsed, while a history of mental illness and lack of social support were rarely endorsed. GPS symptom scores ranged between 0 and 14 (*M* = 5.76; SD = 4.55).

#### 3.2.1 Reliability

Cronbach’s alpha of the GPS symptoms score (*α* = 0.88), PTSD subscale (*α* = 0.81), depression subscale (*α* = 0.82) and dissociation subscale (*α* = 0.76) indicated excellent internal consistency, while the Cronbach’s alpha for subscale disturbances in self-organization (*α* = 0.39) and GAD (*α* = 0.49) was poor. Cronbach’s alpha if item deleted for the GPS symptom score did not indicate any improvements in internal consistency. Inter-item correlations between GPS symptoms were mostly between 0.15 and 0.50 (see Table 2) with some exceptions. GPS item 13 self-harm and item 18 substance abuse showed low correlations with most other GPS items. Item-total correlations were all higher than 0.30 except for item 13 self-harm (*r* = 0.10) and item 18 substance use (*r* = 0.20).

**Table 2:** Inter-item correlations of the GPS items.

	GPS 1	GPS 2	GPS 3	GPS 4	GPS 5	GPS 6	GPS 7	GPS 8	GPS 9	GPS 10	GPS 11	GPS 12	GPS 13	GPS 14	GPS 15	GPS 16	GPS 18
GPS 1. nightmares	1.00	0.68	0.42	0.34	0.47	0.25	0.15	0.23	0.00	0.30	0.20	0.38	0.25	0.45	0.53	0.17	0.33
GPS 2. avoidance	0.68	1.00	0.71	0.44	0.70	0.41	0.41	0.37	0.32	0.48	0.44	0.57	0.18	0.48	0.53	0.20	0.32
GPS 3. hypervigilance	0.42	0.71	1.00	0.48	0.56	0.36	0.38	0.31	0.34	0.45	0.41	0.51	0.19	0.45	0.43	0.27	0.04
GPS 4. numbness	0.34	0.44	0.48	1.00	0.40	0.36	0.19	0.17	0.13	0.34	0.24	0.25	0.07	0.19	0.28	0.31	0.02
GPS 5. Guilt	0.47	0.70	0.56	0.40	1.00	0.43	0.39	0.45	0.40	0.46	0.43	0.65	0.22	0.46	0.60	0.37	0.40
GPS 6. worthless	0.25	0.41	0.36	0.36	0.43	1.00	0.32	0.13	0.40	0.87	0.66	0.30	−0.08	0.50	0.36	0.28	0.27
GPS 7. anger	0.15	0.41	0.38	0.19	0.39	0.32	1.00	0.24	0.34	0.41	0.30	0.53	0.12	0.32	0.27	0.14	0.09
GPS 8. anxiety	0.23	0.37	0.31	0.17	0.45	0.13	0.24	1.00	0.34	0.24	0.27	0.56	0.02	0.31	0.30	0.21	0.14
GPS 9. worrying	0.00	0.32	0.34	0.13	0.40	0.40	0.34	0.34	1.00	0.46	0.56	0.15	−0.05	0.34	0.30	0.29	0.09
GPS 10. depression	0.30	0.48	0.45	0.34	0.46	0.87	0.41	0.24	0.46	1.00	0.71	0.32	−0.09	0.49	0.52	0.28	0.21
GPS 11. anhedonia	0.20	0.44	0.41	0.24	0.43	0.66	0.30	0.27	0.56	0.71	1.00	0.35	−0.09	0.63	0.56	0.38	0.08
GPS 12. insomnia	0.38	0.57	0.51	0.25	0.65	0.30	0.53	0.56	0.15	0.32	0.35	1.00	0.02	0.32	0.38	0.30	0.25
GPS 13. self-harm	0.25	0.18	0.19	0.07	0.22	−0.08	0.12	0.02	−0.05	−0.09	−0.09	0.02	1.00	0.12	−0.10	−0.01	−0.05
GPS 14. derealization	0.45	0.48	0.45	0.19	0.46	0.50	0.32	0.31	0.34	0.49	0.63	0.32	0.12	1.00	0.68	0.28	0.21
GPS 15. depersonalization	0.53	0.53	0.43	0.28	0.60	0.36	0.27	0.30	0.30	0.52	0.56	0.38	−0.10	0.68	1.00	0.27	0.30
GPS 16. other problems	0.17	0.20	0.27	0.31	0.37	0.28	0.14	0.21	0.29	0.28	0.38	0.30	−0.01	0.28	0.27	1.00	−0.02
GPS 18. substance abuse	0.33	0.32	0.04	0.02	0.40	0.27	0.09	0.14	0.09	0.21	0.08	0.25	−0.05	0.21	0.30	−0.02	1.00

Inter-item correlations between 0.15 and 0.50 were considered adequate. GPS, Global psychotrauma screen.

#### 3.2.2 Clinical validity

Based on the CAPS-5, 24 participants (34.3%) met the criteria for a DSM-5 PTSD diagnosis and 21 participants (30.0%) met the criteria for dissociation. In total, 17 participants met criteria for both PTSD and dissociation and therefore met criteria for the dissociative subtype. Based on the SCID-5, 12 participants (17.1%) met the criteria for a major depressive disorder (MDD), and only one person (1.5%) met the criteria for GAD. Given the low GAD occurrence, we were unable to test the clinical validity of the GPS for indicating a probable GAD. Table 3 presents the clinical validity of the GPS using the Youden index to provide optimal cut-off points for PTSD, MDD and dissociation.

**Table 3:** Clinical validity characteristics of the GPS for PTSD, MDD and Dissociation.

Construct	Optimal cut-off	Sensitivity (95% CI)	Specificity (95% CI)	PPV (95% CI)	NPV (95% CI)	Positive DLR	Negative DLR	AUC	Youden index
PTSD	3	1.00 (0.85-NA)	0.76 (0.61–0.87)	0.68 (0.51-NA)	1.00 (0.89–1.00)	4.18 (2.49–7.00)	0.00	0.91	0.76
MDD	1	1.00 (0.73-NA)	0.72 (0.58–0.83)	0.44 (0.30-NA)	1.00 (0.90–1.00)	3.60 (2.34–5.53)	0.00	0.90	0.72
Dissociation	1	0.95 (0.76–0.99)	0.95 (0.83–0.99)	0.90 (0.72–0.99)	0.97 (0.88–0.99)	23.33 (5.98–90.90)	0.04	0.96	0.90

**Figure 2:**
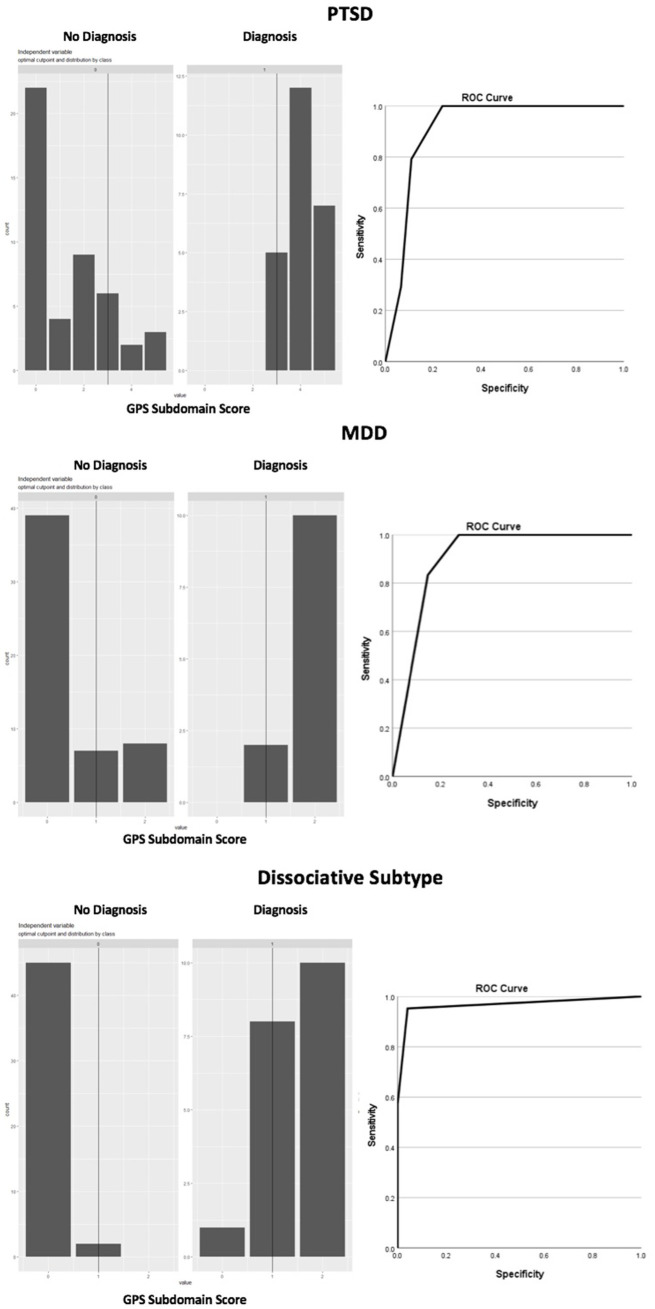
GPS subscale scores.

A GPS PTSD subscale score of 3 or higher was optimally efficient for detecting probable PTSD (Youden’s *J* = 0.76) with a sensitivity of 1.00 (95% CI: 0.85-NA) and a specificity of 0.76 (95% CI: 0.61–0.87). A GPS subscale score of 1 or higher was optimally efficient for detecting probable dissociation (Youden’s *J* = 0.90), with a sensitivity of 0.95 (0.76–0.99), and specificity of 0.95 (0.86–0.99). These cut-offs for probable PTSD and dissociation resulted in a combined indication for the dissociative subtype. This indication had a high accuracy (91%), a sensitivity of 0.94 (95% CI: 0.71–1), and specificity of 0.91 (95% CI: 0.79–0.97). A GPS MDD subscale score of 1 or higher was optimally efficient for detecting probable MDD (Youden’s *J* = 0.72), with a sensitivity of 1.00 (95% CI: 0.73-NA), and a specificity of 0.72 (95% CI, 0.58–0.83). Figure 2 depicts the GPS subscale scores for true positives and true negatives including the cut-off point for PTSD, MDD and dissociation and depicts the ROC curves.

#### 3.2.3 Convergent validity

Regarding the convergent validity of the GPS resilience item, we found that it was not significantly related to the BRS total score (*r* = 0.02, *p* = 0.87). Moreover, regarding the relationship between risk factors of the GPS and GPS symptom scores, linear regression analyses showed that current stressors [*b* = 3.80, *t*(63) = 3.52, *p* = 0.001] and childhood trauma [*b* = 4.51, *t*(63) = 4.42, *p* < 0.001] were significantly related to higher GPS symptom scores, while lack of resilience (*p* = 0.08), lack of social support (*p* = 0.28), and history of mental illness (*p* = 0.19) were not.

## 4 Discussion

This study examined the reliability, clinical and convergent validity of the English version of the GPS (Olff et al., 2020) in a sample of war-impacted African refugees resettled in Australia aged 18 and older. Our results provide preliminary support for the GPS as a useful screening tool to detect probable PTSD, MDD and the dissociative subtype. To our knowledge, this is the first study to validate a transdiagnostic posttraumatic screening measure inclusive of risk and protective factors in a clinical sample of refugee populations, and the first study of the GPS using clinical interviews to determine the clinical validity.

### 4.1 Reliability

Our results indicated that the GPS symptoms score, reflecting the transdiagnostic overall symptom burden, in addition to the GPS subscales that screen for PTSD, dissociation and MDD were reliable, while for GAD and DSO this was not found. GPS validation studies in other populations also generally found that subscales GAD and DSO showed a relatively low internal consistency (e.g., Oe et al., 2020). Both constructs are only assessed with two items, which might be insufficient for a reliable measurement of these constructs. Future studies might assess whether adding more items for these constructs might improve the reliability. However, the main purpose of the GPS is to efficiently screen for probable trauma-related disorders, and to keep the items to a minimum. The present study also documented that substance abuse and self-harm had low inter-item correlations with other GPS symptoms, while self-harm also showed low item-total correlations. Both constructs were rarely endorsed in the current sample compared to previous validation studies and to recent norm data (Oe et al., 2020; Frewen et al., 2021; Rossi et al., 2021; Global Collaboration on Traumatic Stress, 2022). This might be related to the current population (African refugees), since cultural meaning, functions, and manifestation of symptoms such as self-harm might differ cross-culturally (Gargiulo et al., 2021). Moreover, symptoms may differ within cultural groups. It is plausible that the present sample, consisting of 78.5% adults with an active occupation (employed or studying) may be less prone to substance misuse compared to marginalized African refugee youth in Australia (Horyniak et al., 2016; Mwanri and Mude, 2021) which have been prominently documented to abuse alcohol. Likewise, higher ratings among asylum seekers in Australia have been documented among those in off-shore detention centers compared to those in community-based arrangements (Hedrick et al., 2019). Further studies are needed to further investigate the prevalence of self-harm and substance abuse among representative African refugee populations.

### 4.2 Clinical validity

We found that the GPS presented adequate efficiency with moderately high sensitivity and specificity for detecting a probable diagnosis of PTSD, dissociation, dissociative subtype and MDD, but we were unable to test this for GAD. The area under the curve for PTSD, dissociation and MDD was above 0.90, reflecting an excellent screener ability of the GPS in this population (Youngstrom, 2014). A clinical cut-off score of 3 and higher on the GPS PTSD subscale was optimal for detecting probable PTSD, and a cut-off score of 1 or higher was optimal on the GPS subscales dissociation and MDD for detecting probable dissociation and MDD, respectively. The sensitivity and specificity for PSTD, dissociation, the dissociative subtype, and MDD documented in this study demonstrate adequate accuracy of the GPS in screening for probable clinical diagnoses assessed with the CAPS-5 and SCID-5. The sensitivity was very high for all these outcomes, indicating that people suffering from the disorder may screen positive on the GPS. For a screening instrument, it is purposeful to be optimally sensitive and thus have rare false negative screenings, as the aim is not to miss potential trauma survivors with these problems. Positive screenings are usually followed-up by a clinical interview, where potential false positives may be detected. Specificity for MDD, and to lesser extend for PTSD, was moderately high, underscoring the importance of follow-up assessments in clinical settings. As noted, validity analyses for GAD were not possible due to the low GAD occurrence in this sample (1.5%) according to the SCID-5. This low endorsement may be explained by the SCID-5 GAD criteria F, “the disturbance is not better explained by another mental disorder” (First et al., 2015b).

The relevance of these findings is inherently aligned with the advantages of screening over diagnostic tests. Trauma survivors may suffer from a wide range of disorders and problems, as well as comorbid presentations, that would require a large test battery to detect. This benefits both the individual and the healthcare system, for instance “placing fewer demands on the healthcare system and being more accessible as well as less invasive, less dangerous, less expensive, less time-consuming, and less physically and psychologically discomforting for clients” (Trevethan, 2017). These advantages become particularly valuable for trauma-impacted populations in low-resource settings. Compared to existing screening measures, the GPS presents the unique transdiagnostic quality in a simple and brief format, suitable for both research and clinical settings.

### 4.3 Risk and protective factors

We also examined associations between the GPS symptom scores and risk and protective factors. We found that current stressors and childhood trauma were related to higher GPS symptom scores, while lack of resilience, social support, and history of mental illness were not significantly associated with the GPS symptom scores. Lack of social support, and a history of mental illness, were both only endorsed by a few people, much less compared to previous GPS validation studies in other samples (Oe et al., 2020; Frewen et al., 2021; Rossi et al., 2021). While speculative, we suggest that the low mental illness history endorsement may be explained by a lack of awareness rather than a lack of illness, given the low accessibility to mental health professionals in the participants’ home countries. As addressed in the pilot test of the Japanese version of the GPS, cross-cultural differences may exist in response to traumatic experiences, including underreporting and stigma (Oe et al., 2020). In support, studies with samples of Sudanese refugees in Australia have indicated stigma (Savic et al., 2016) and low mental health literacy levels (May et al., 2014) to be prevalent, but the same is not known for Liberian and Congolese populations.

Lastly, we examined the convergent validity of the GPS resilience item concerning the BRS total score and found non-significant results. Challenges with the BRS scale have been previously reported in another study with refugees, indicating potential inadequacy in the BRS index and contextual nuances when assessing resilience cross-culturally (Tay et al., 2021). Alternatively, one item measuring psychological resilience might not be sufficient for such a complex construct. Future studies might correlate the GPS resilience item with other measurements of resilience such as the Resilience Evaluation Scale (van der Meer et al., 2018).

### 4.4 Limitations

This study presented some limitations. First, since the data was collected as part of a larger treatment trial, the enrolment criteria involved excluding participants with substance abuse. Moreover, while the study sample presents a reasonably balanced gender ratio (*M* = 31, *F* = 39), of those who fulfilled the PTSD criteria, 65.7% of the participants were female. This gender imbalance has been documented among displaced African women (Idemudia et al., 2014) and is consistent with the previous evidence of women being at greater risk for developing posttraumatic stress outcomes, including PTSD (Olff et al., 2007; Olff, 2017; Ramikie and Ressler, 2018). Although the year range of trauma exposure in the inclusion criteria was extensive to accommodate refugees exposed to war and conflict across 3 countries at different times, it is possible that age at onset and number of years in resettlement may have impacted severity levels, alongside post-migration difficulties most associated with DSO and CPTSD. The validity indices presented for the SCID-5, CAPS-5, and BRS were obtained from western populations and not representative of refugees and Sub-Saharan Africans. While a strength of the study is the use of golden standard clinical interviews to assess diagnoses of all axis I psychiatric conditions, few or no endorsements in relevant disorders, such as GAD and sleep disorders, limited the present analyses.

### 4.5 Future directions

Future studies with larger samples are recommended to establish the usability of the GPS as an efficient screening tool for trauma-related disorders. In particular, studies are needed to investigate the validity of the GPS in screening anxiety, self-organization, and resilience among refugees. Moreover, while participants’ English language skills were satisfactory for the present study, new studies validating translated versions of the screen in languages widely spoken by underrepresented populations across conflict-impacted regions would be valuable. Considering the present results are based on English-speaking African refugees resettled in a developed country, the generalizability of the present results to global refugee populations, including culturally diverse groups, requires caution and further research. While gender disparities may be challenging to address in future studies, methods should be designed to ensure gender-sensitive analyses (Olff, 2019). Lastly, considering the difficulties faced with the BRS instrument, future studies should cautiously examine the cultural appropriateness of resilience (and other) measures for refugee populations, particularly in the absence of normative data.

## 5 Conclusion

This study is the first to examine the psychometric properties of the GPS among a subclinical sample of traumatized adults, and to first document the validity of a trauma-focused transdiagnostic screening tool for refugee populations. The exponential growth of refugee numbers, surpassing 100 million worldwide (United Nations High Commissioner for Refugees, 2022), has created a pressing need in the humanitarian sector for a brief and transdiagnostic screening measure inclusive of the diverse outcomes of traumatic exposure. We conclude that the GPS demonstrated excellent internal consistency, sensitivity, and specificity to detect probable PTSD, MDD, and the dissociative subtype, and may be a useful transdiagnostic screening tool for refugees.

The GPS is freely available and accessible online^5^ in over 30 languages.

## Data availability statement

The datasets presented in this study can be found in online repositories. The names of the repository/repositories and accession number(s) can be found at: https://osf.io/ehs62/?view_only=10b330f0e2c94a6b9b2995fa8aa824d5.

## Ethics statement

The study was approved by the University of Sydney Human Research Ethics Office and was conducted in compliance with ethics committee approval conditions. All participants voluntarily provided oral and written informed consent.

## Author contributions

JP: Conceptualization, Data curation, Funding acquisition, Investigation, Methodology, Project administration, Resources, Visualization, Writing – original draft, Writing – review & editing, Validation. ChH: Data curation, Formal analysis, Methodology, Supervision, Validation, Visualization, Writing – original draft. CaH: Methodology, Supervision, Validation, Visualization, Writing – review & editing. BO’T: Methodology, Supervision, Validation, Visualization, Writing – review & editing. MO: Supervision, Validation, Visualization, Writing – review & editing.

## Footnotes

^1^ https://www.traumameasuresglobal.com/itq

^2^Single measure of the combination of sensitivity and specificity (Sensitivity + Specificity – 1) which has a value between 0 and 1.

^3^Ratio of true positive diagnoses.

^4^Ratio of true negative diagnoses.

^5^ www.global-psychotrauma.net/gps
